# Supplementary material for: NLRP3 inflammasome in fibroblasts links tissue damage with inflammation in breast cancer progression and metastasis
Source: Nat Commun. 2019 Sep 26;10:4375. doi: 10.1038/s41467-019-12370-8 (PMC6763472; doi:10.1038/s41467-019-12370-8)
Supplement: Supplementary file 1 — Supplementary Info [file 41467_2019_12370_MOESM1_ESM.pdf]

Supplementary Information for:

NLRP3 inflammasome in fibroblasts links tissue damage with inflammation in breast cancer progression and metastasis

Ershaid *et al.*

This PDF includes:

Supplementary Figures 1-7 and Legends

Supplementary Table 1

Supplementary Fig. 1

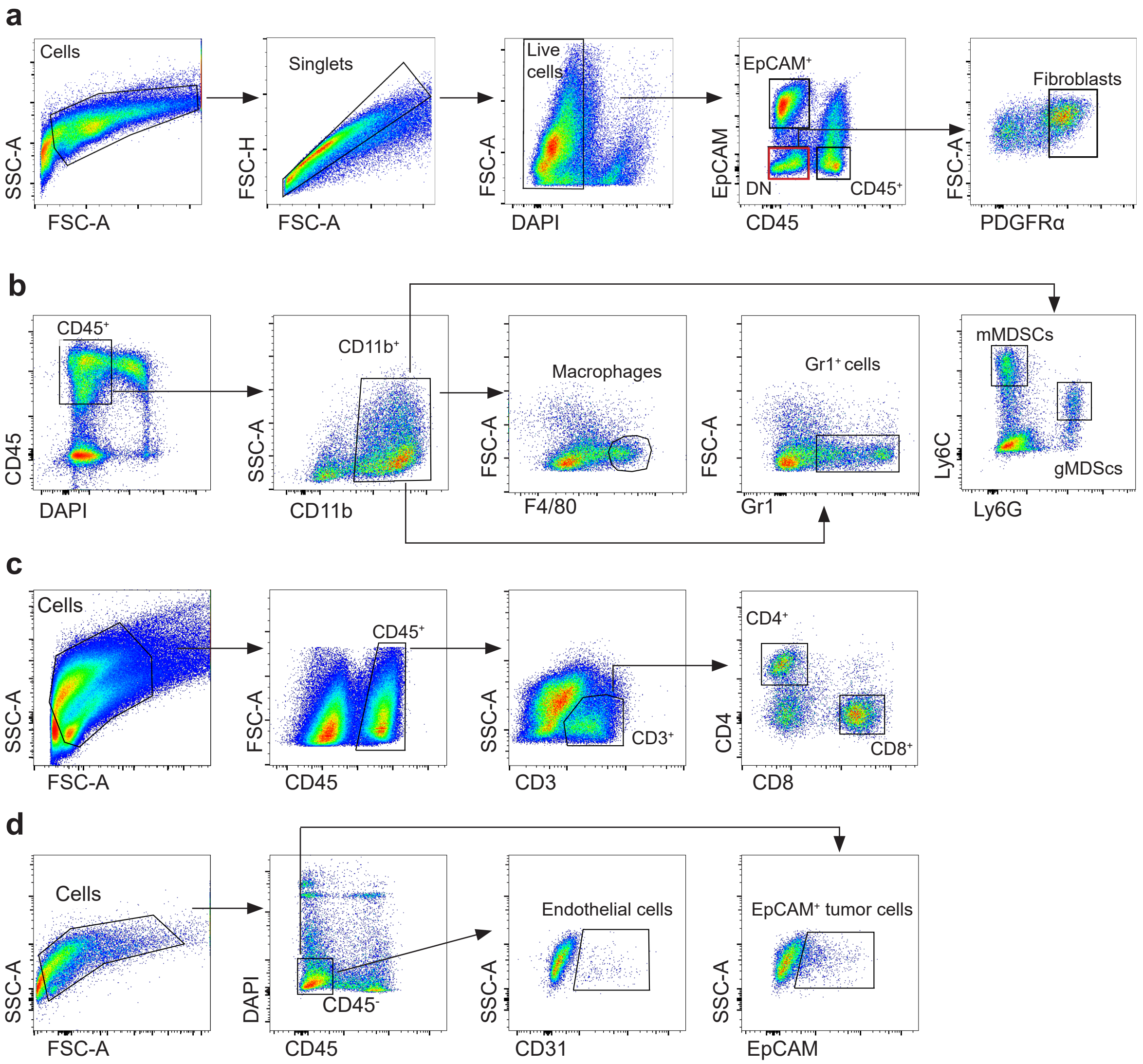

Supplementary Fig. 1 Gating strategies used for flow cytometry sorting and analysis (**a**) Gating strategy to sort normal fibroblasts or CAFs for qRT-PCR analysis of inflammasome pathway expression presented in Fig. 1f. The same gating strategy was used to analyze NLRP3 expression in normal fibroblasts or CAFs (Supplementary Fig. 1b,c). (**b**) Gating strategy to used for analysis of tissue infiltration of total myeloid cells (CD11b^{+}) cells, macrophages (CD11b^{+}F4/80^{+} cells), total MDSCs (CD11b^{+}Gr1^{+}) cells, mMDSC (CD11b^{+}Ly-6C^{high}Ly6G^{-}), and gMDSCs (CD11b^{+}Ly6C^{low}Ly6G^{+}) shown in Fig. 4d,g,h,l-n, Fig.5i-l, Supplementary Fig. 4d,e Supplementary Fig. 5e,f, and Supplementary Fig. 6c,d,h. The same gating strategy was used to sort mMDSCs and gMDSCs for qRT-PCR analysis of immune-supression markers presented in Supplementary Fig. 6a-l. (**c**) Gating strategy used for analysis of tissue infiltration of CD3 lymphocytes (CD3^{+}) cells, CD4 lymphocytes (CD3^{+}/CD4^{+} cells), and CD8 lymphocytes (CD3^{+}CD8^{+}) shown in Supplementary Fig. 4f-h, Supplementary Fig. 5g-i, and Supplementary Fig. 6e-g,i-k. (**d**) Gating strategy to sort endothelial cells and tumor cells for qRT-PCR analysis of the expression of adhesion-related genes (Fig. 6e,f) and the expression of invasion-related genes (Fig. 6g), respectively.

Supplementary Fig. 2 (related to Fig. 1)

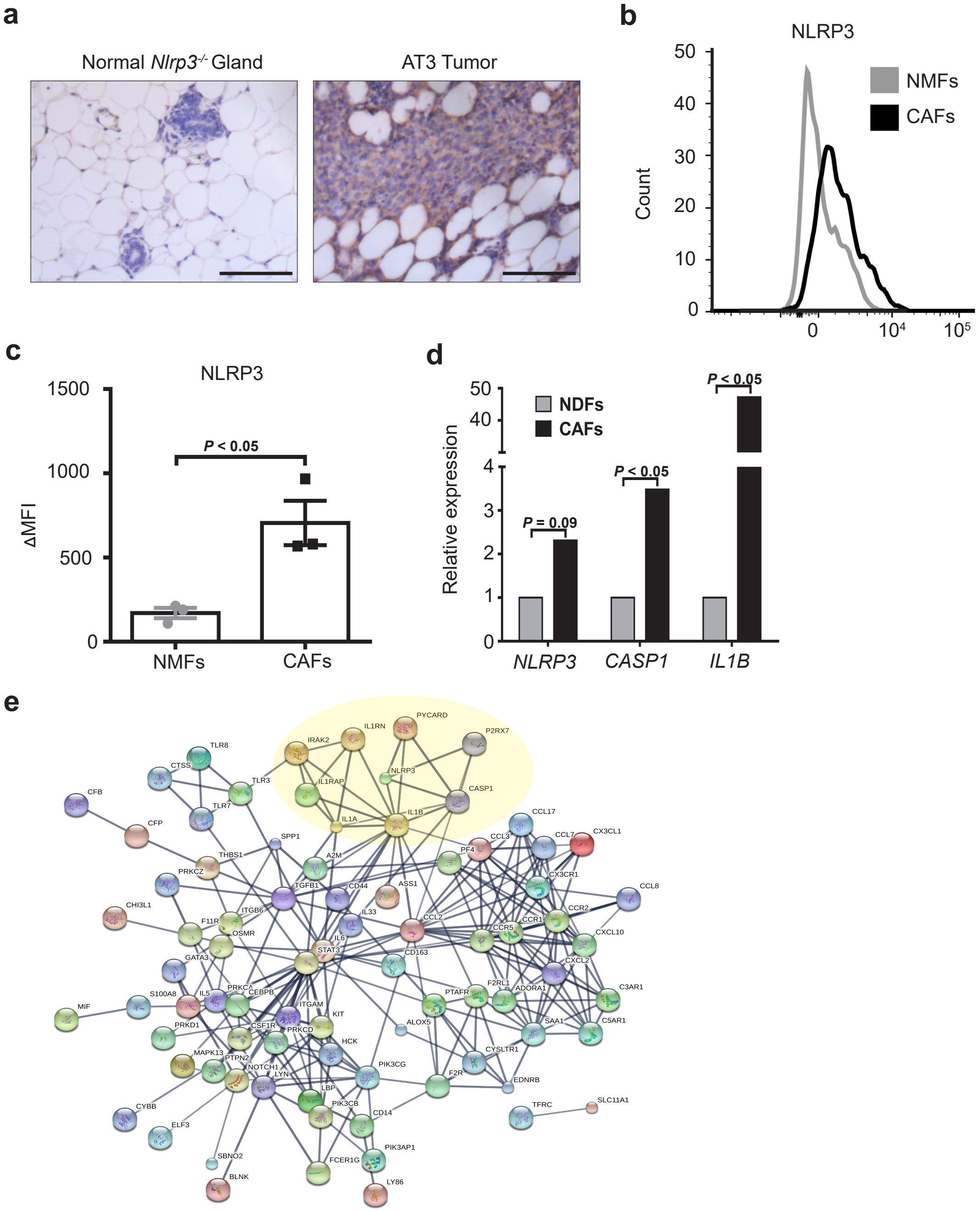

Supplementary Fig. 2 (**a**) Representative IHC staining of NLRP3 in normal mammary glands of *Nlrp3*$^{-/-}$ mice or of AT3 mamamry tumors. Scale bar, 100 μm. (**b**) Flow cytometry analysis of NLRP3 levels in fibroblasts isolated from normal mammary glands (NMFs) or isolated from mammary tumors (CAFs). (**c**) ΔMFI values representing NLRP3 expression levels were quantified. n= 3 pools of 3 mice/group. Data are represented as mean ±s.e.m; Welch's t-test. Representative of two independent experiments. (**d**) Expression levels of *Nlrp3/Il1b* pathway related genes in CAFs of squamous cell carcinoma of the skin or in normal dermal fibroblasts (NDFs). Data was obtained from NCBI GEO (Dataset accession number GSE17817); Welch's t-test. (**e**) Pro-inflammatory genes that were upregulated in mammary CAFs isolated from murine advanced carcinoma (upregulated at least 1.4 fold as compared with normal mammary glands), were analyzed using the STRING database for human protein-protein interactions. Genes related to the NLRP3 inflammasome are highlighted. Edge Confidence>0.7. Source data are provided as a Source Data file.

Supplementary Fig. 3 (related to Fig. 2 & Fig.3)

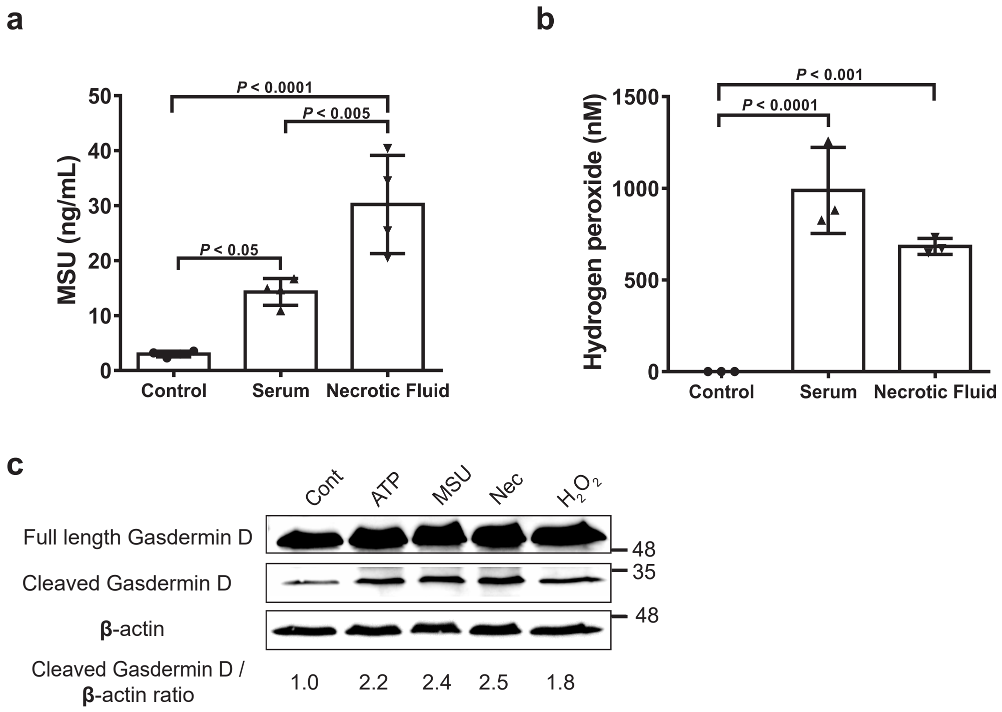

Supplementary Fig. 3 Levels of (**a**) monosodium urate crystals or (**b**) hydrogen peroxide in necrotic fluid extracted from PyMT tumors or in plasma of FVB/n mice were detected using commercial detection kits. n=3-4 mice/group. Data are represented as mean ± s.d; One-way analysis of variance test followed by Tukey's multiple comparisons test. (**c**) Gasdermin D processing was assessed by western blot of cell lysates with anti-Gasdermin D antibody. β-actin was utilized as a loading control. The samples derive from the same experiment. Data are representative of 2 independent experiments. Source data are provided as a Source Data file.

Supplementary Fig. 4 (related to Fig. 4)

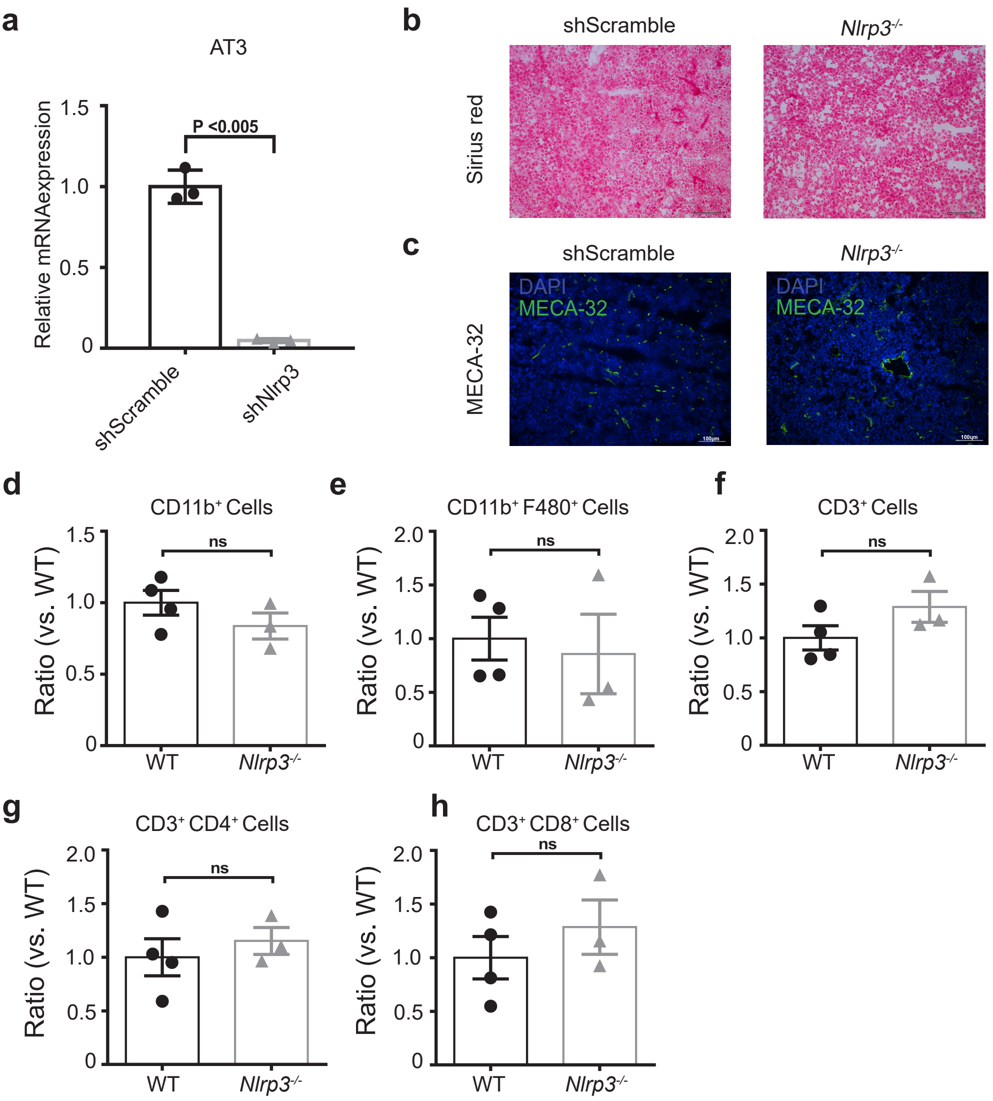

Supplementary Fig. 4 (**a**) qRT-PCR analysis of *Nlrp3* expression in AT3 cells transduced with scramble shRNA (shScramble) or *Nlrp3*-targeting shRNA (shNlrp3). Data are represented as mean ± s.d; Welch's t-test. (**b**) Representative images of Sirius red staining of AT3 tumors. n=6 tumors per group. Scale bar, 100μm. (**c**) Representative images of immunofluorescent staining with anti-Meca32 antibody in AT3 tumors. n=6 tumors per group. Scale bar, 100μm. (**d-e**) Flow cytometry analysis of immune cell infiltration into AT3 tumors co-injected with WT (*Nlrp3*$^{+/+}$) fibroblasts or *Nlrp3*$^{-/-}$ fibroblasts. Data presented are percentage of CD45^{+} cells, normalized to WT. n=4 and 3 tumors per group (WT and *Nlrp3*$^{-/-}$, respectively). Representative of three independent experiments. Data are represented as mean ± s.e.m; One-way analysis of variance test followed by Tukey's multiple comparisons test. Source data are provided as a Source Data file.

Supplementary Fig. 5 (related to Fig. 4)

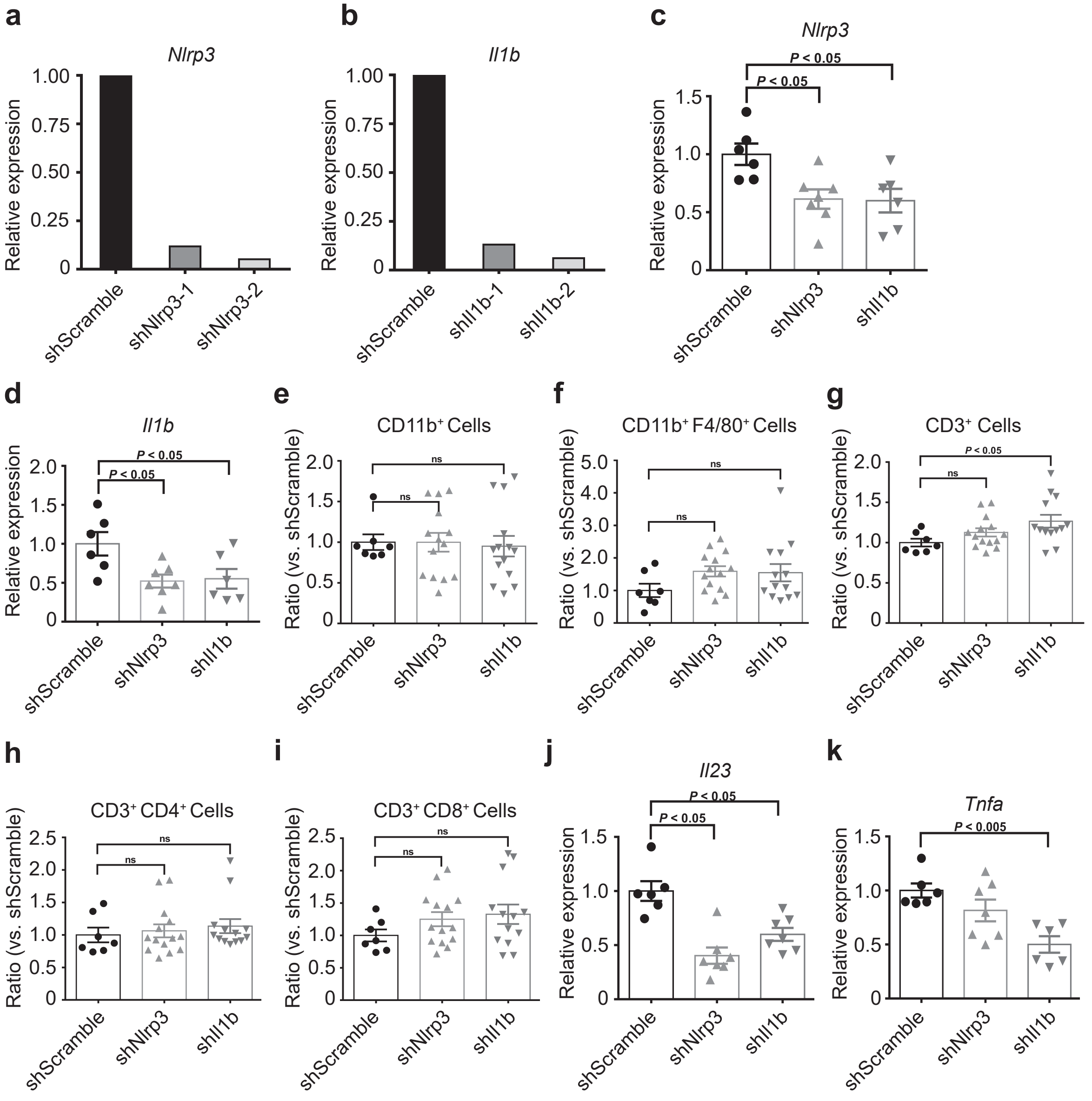

Supplementary Fig. 5 (**a,b**) qRT-PCR analysis of (a) *Nlrp3 or (b) Il1b* expression in mammary fibroblasts transduced with scramble shRNA (shScramble), *Nlrp3*-targeting shRNA (shNlrp3-1, shNlrp3-2), or *Il1b*-targeting shRNA (shIl1b-1, shIl1b-2). (**c,d**) Met-1 tumors were analyzed for the expression of (c) *Nrp3* and (d) *Il1b* using qRT-PCR. n= 6, 7, and 6 individual tumors per group (shScramble, shNlrp3, and shIl1b, respectively). Data are presented as fold change from shScramble. Representative of three independent experiments. (**e-i**) Flow cytometry analysis of immune cell infiltration into Met-1 tumors co-injected with fibroblasts silenced for *Nlrp3* or *Il1b* (shNlrp3, shIl1b) or control fibroblasts (shScramble). Data presented are percentage of CD45$^+$ cells, normalized to shScramble. n=7, 14, and 13 tumors per group (shScramble, shNlrp3, shIl1b, respectively). One-way analysis of variance test followed by Tukey's multiple comparisons test. Representative of three independent experiments. (**j,k**) Met-1 tumors were analyzed for the expression of *Il23* (j) and *Tnfa* (k) using qRT-PCR. n= 6, 7, and 6 individual tumors per group (shScramble, shNlrp3, and shIl1b, respectively). In (**c**, **d**, **j**, **k**) data are represented as mean ± s.e.m; One-way analysis of variance test followed by Dunnett's multiple comparisons test. Source data are provided as a Source Data file.

Supplementary Fig. 6 (related to Fig. 5)

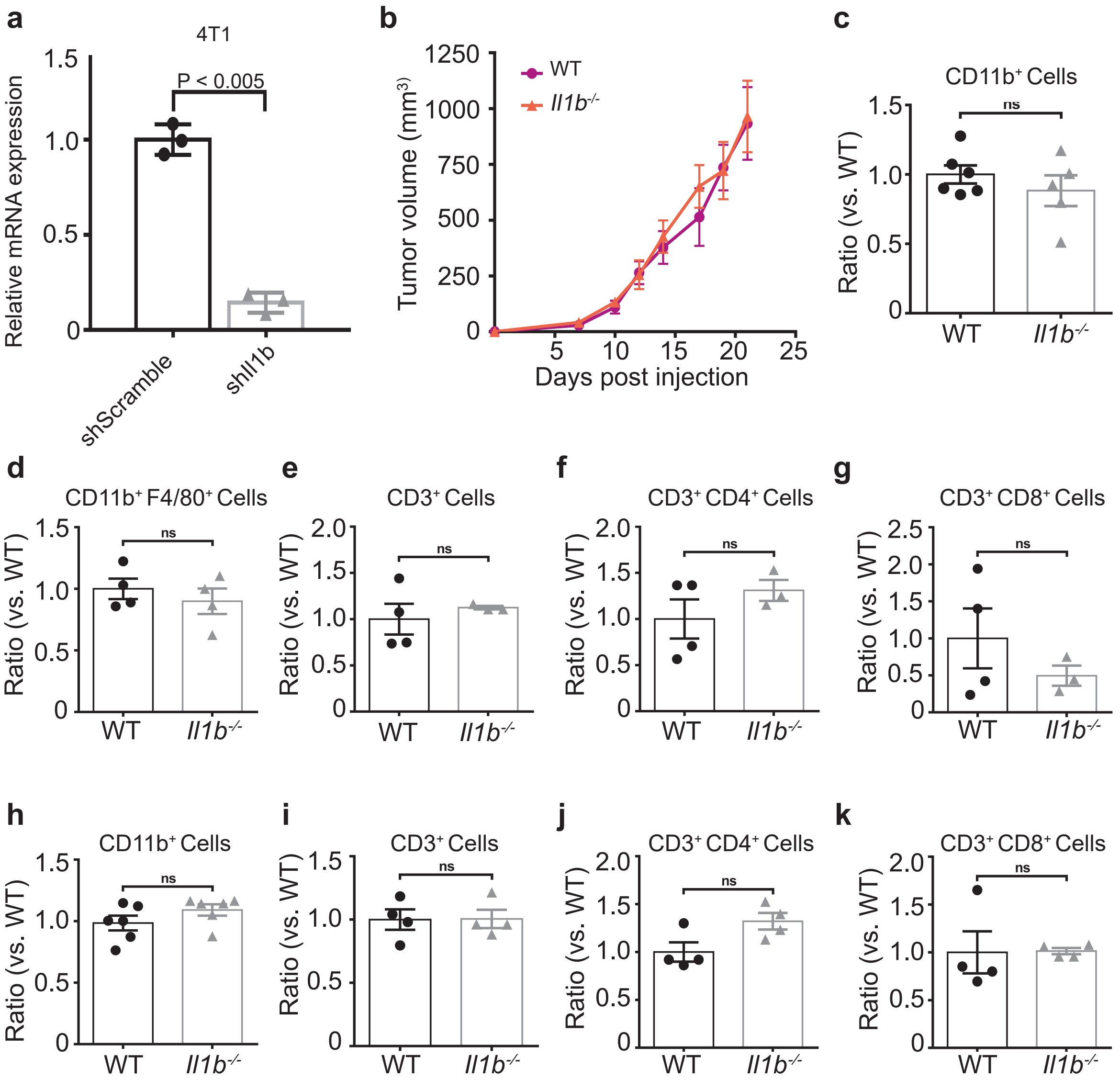

Supplementary Fig. 6 (**a**) qRT-PCR analysis of Il1b expression in 4T1 cells transduced with scramble shRNA (shScramble) or Il1b -targeting shRNA (shIl1b). Error bars represent s.d of technical repeats. Welch's t-test *,p<0.05 (**b**) Growth curves of 4T1 tumors co-injected with WT NMFs (*Il1b*$^{+/+}$) or with *Il1b*$^{-/-}$ NMFs into mammary glands of WT mice. n=5 and 7 individual tumors per group (WT and *Il1b*$^{-/-}$,respectively) . Representative of three independent experiments. Data are represented as mean ±s.e.m; Welch's t-test. (**c-g**) Flow cytometry analysis of immune cell infiltration into 4T1 tumors co-injected with WT fibroblasts (*Il1b*$^{+/+}$) or *Il1b*$^{-/-}$ fibroblasts. In (**c**) n=6 and 5 individual tumors per group. (WT, and *Il1b*$^{-/-}$ respectively). In (**d**) n= 4 tumors per group. In (**e-g**) n=4 and 3 (WT, and *Il1b*$^{-/-}$ respectively). (**h-k**) Flow cytometry analysis of immune cell infiltration into lungs of 4T1-tumors bearing mice. In (**h**) n=6 per individual mice per group. In (**i-k**) n=4 individual tumors per group. In (**c-k**) data presented are percentage of CD45^{+} cells, normalized to WT. Data are represented as mean ± s.e.m; Welch's t-test. Representatives of 3 independent experiments. Source data are provided as a Source Data file.

Supplementary Fig. 7 (related to Fig. 5)

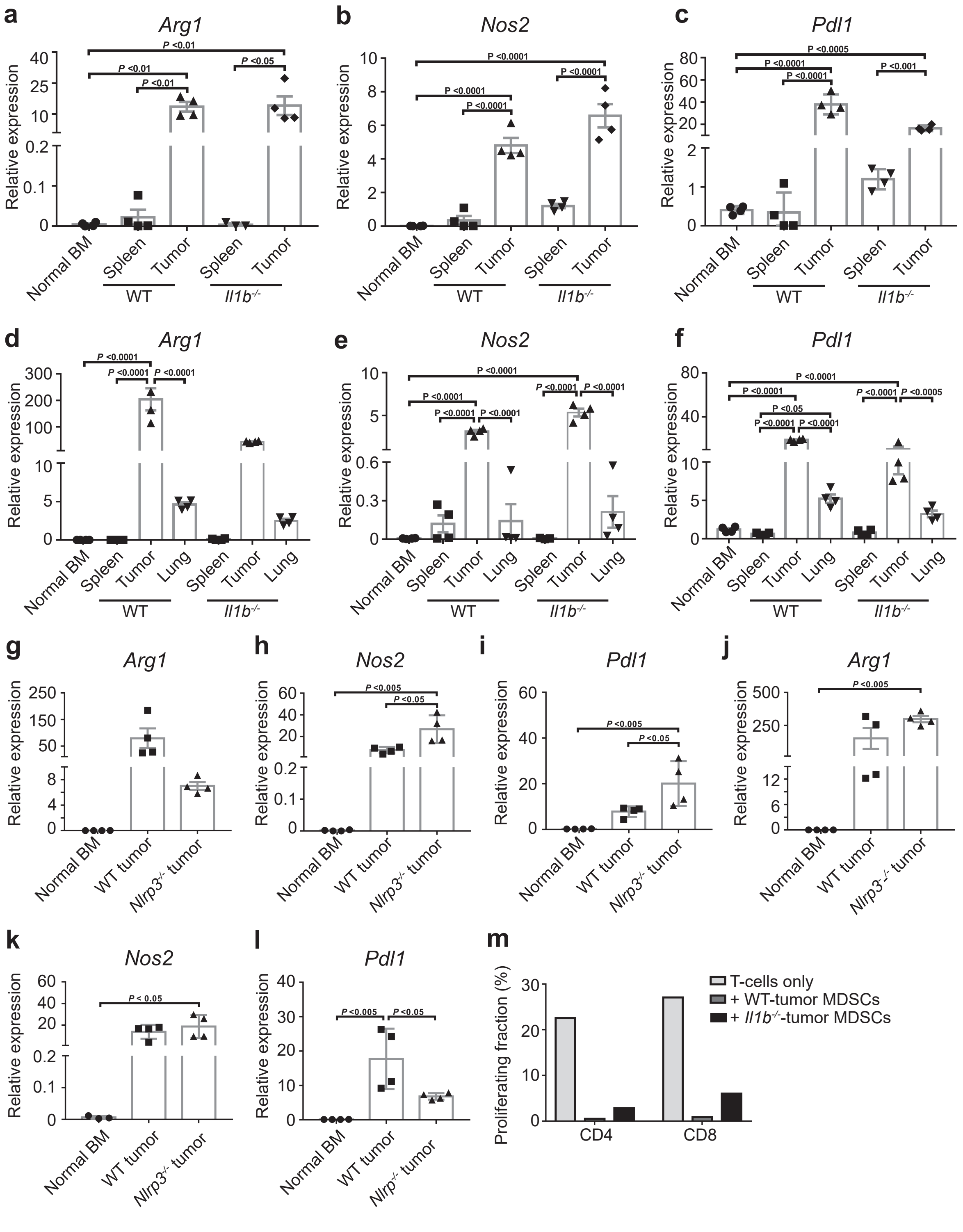

Supplementary Fig. 7 (**a-f**) qRT-PCR analysis of immune-suppression markers in CD11b$^+$Ly6G$^+$Ly6C^{low} cells (**a-c**) or in CD11b$^+$Ly6C^{high}Ly6G$^-$ cells (**d-f**), FACS sorted from 4T1-tumor bearing mice injected with WT fibroblasts (*Il1b*$^{+/+}$) or *Il1b*$^{-/-}$ fibroblasts or from bone marrow of tumor-free mice. Data are presented as mean ± s.e.m of 4 mice per group; One-way analysis of variance test followed by Tukey's multiple comparisons test. (**g-l**) qRT-PCR analysis of markers of immune-suppression markers in (**g-i**) CD11b$^+$ CD11b$^+$Ly6G$^+$Ly6C^{low} cells or in (**j-l**) CD11b$^+$Ly6C^{high}Ly6G$^-$ cells sorted from AT3 tumors co-injected with WT fibroblasts (*Nlrp3*$^{+/+}$) or *Nlrp3*$^{-/-}$ fibroblasts or from bone marrow of tumor-free mice. Data are presented as mean ± s.e.m of 4 mice per group; One-way analysis of variance test followed by Tukey's multiple comparisons test. (**m**) CFSE assay to assess suppressive activity of CD11b$^+$ Gr1$^+$ cells FACS-sorted from 4T1-tumors injected with WT (*Il1b*$^{+/+}$) or *Il1b*$^{-/-}$ fibroblasts on CD4$^+$ or CD8$^+$ splenocytes. Proliferation was assessed by dilution of CFSE dye. Source data are provided as a Source Data file.

Supplementary Table 1: List of primers

Gene	Forward primer sequence	Reverse primer sequence
Acta2	AGCCAGTCGCTGTCAGGAA	CGAAGCCGGCCTTACAGA
Adam12	AGACGTGCTGACTGTGCAAC	CCGTGTGATTTCGAGTGAGAGA
Arg1	GGAATCTGCATGGGCAACCTGTGT	AGGGTCTACGTCTCGCAAGCCA
Casp1	ACTGCCTGCTGCTTCTCCTACA	ACAAGACCAGGCATATTCTTTCATG
Ccl3	GCTTTCTGCTCTTCAACACCAGATA	AGGAAAATGACACCTGGCTGG
Cxcl2	CATCCAAAAGATACTGAACAAAGGC	TTTCTCTTTGGTTCTTCCGTTGA
Gapdh	TCTTGTGCAGTGCCAGCCT	CCAATACGGCCAAATCCGT
Gusb	GACTGGAAGCTTGGGGCTTA	GTCCCCATAGCTCCTGAGACCT
Il1a	TTAAATGACCTGCAACAGGAAGTAA	GCTCACGAACAGTTGTGAATCTG
Il1b	ACCCCAAAAGATGAAGGGCT	GATACTGCCTGCCTGAAGCTCT
Il18	CGACTTCACTGTACAACCGCAGT	GTCTGGGGTTCACTGGCACT
Il1r2	TTTAAATGTGTTGCCTCGAATCC	CCAGGAGAACGTGGAAGAGACT
Il6	ATACCACTCCCAACAGACCTGTCT	CAGAATTGCCATTGCACAACTC
Il23	GCACCAGCGGGACATATGAA	CTGGCTGTTGTCCTTGAGTCCT
Icam1	GTGATGCTCAGGTATCCATCCA	CACAGTTCTCAAAGCACAGCG
Mmp1a	AACTACATTTAGGGGAGAGGTGT	GCAGCGTCAAGTTTAACTGGAA
Mmp3	ACATGGAGACTTTGTCCCTTTTG	TTGGCTGAGTGGTAGAGTCCC
Mmp10	GAGCCACTAGCCATCCTGG	CTGAGCAAGATCCATGCTTGG
Nlrp3	CCCTTGGAGACACAGGACTCA	TGAGGCTGCAGTTGTCTAATTCC
Nos2	TTCACCCAGTTGTGCATCGACCTA	TCCATGGTCACCTCCAACACAAGA
P2rx7	CAAAGGCCAAGAAGTTCCAAGA	TCTTCATGGAGCAGCTGAATTTC
Pdl1	AACACATCCTCCACAGAACAG	CGGTGAATGTTTCAGATTGGAGT
Sele	ATGAAGCCAGTGCATACTGTC	ACCCGTGAGTTATTCCATGAGT
Selp	CATCTGGTTCAGTGCTTTGATCT	ACCCGTGAGTTATTCCATGAGT
Spp1	GGAGGAAACCAGCCAAGGAC	GGGAGGAGGCAATGCCAA
Ubc	GCCCAGTGTTACCACCAAGA	CCCATCACACCCAAGAACA
Vcam1	TTGGGAGCCTCAACGGTACT	GCAATCGTTTTGTATTCAGGGGA
